# Honey for Wound Healing, Ulcers, and Burns; Data Supporting Its Use in Clinical Practice

**DOI:** 10.1100/tsw.2011.78

**Published:** 2011-04-05

**Authors:** Noori Al-Waili, Khelod Salom, Ahmad A. Al-Ghamdi

**Affiliations:** ^1^ Al-Waili's Foundation for Sciences, Chronic Wound Management and Hyperbaric Medicine, Life Support Technology Group, New York, USA; ^2^ Bee Research Chair, King Saud University, Riyadh, Saudi Arabia

**Keywords:** honey, wound, ulcer, healing, infection, nitric oxide, prostaglandin

## Abstract

The widespread existence of unhealed wounds, ulcers, and burns has a great impact on public health and economy. Many interventions, including new medications and technologies, are being used to help achieve significant wound healing and to eliminate infections. Therefore, to find an intervention that has both therapeutic effect on the healing process and the ability to kill microbes is of great value. Honey is a natural product that has been recently introduced in modern medical practice. Honey's antibacterial properties and its effects on wound healing have been thoroughly investigated. Laboratory studies and clinical trials have shown that honey is an effective broad-spectrum antibacterial agent. This paper reviews data that support the effectiveness of natural honey in wound healing and its ability to sterilize infected wounds. Studies on the therapeutic effects of honey collected in different geographical areas on skin wounds, skin and gastric ulcers, and burns are reviewed and mechanisms of action are discussed. (Ulcers and burns are included as an example of challenging wounds.) The data show that the wound healing properties of honey include stimulation of tissue growth, enhanced epithelialization, and minimized scar formation. These effects are ascribed to honey's acidity, hydrogen peroxide content, osmotic effect, nutritional and antioxidant contents, stimulation of immunity, and to unidentified compounds. Prostaglandins and nitric oxide play a major role in inflammation, microbial killing, and the healing process. Honey was found to lower prostaglandin levels and elevate nitric oxide end products. These properties might help to explain some biological and therapeutic properties of honey, particularly as an antibacterial agent or wound healer. The data presented here demonstrate that honeys from different geographical areas have considerable therapeutic effects on chronic wounds, ulcers, and burns. The results encourage the use of honey in clinical practice as a natural and safe wound healer.

### PATHOPHYSIOLOGY OF WOUND HEALING

Every year in the U.S., more than 1.25 million people have burns and 6.5 million have chronic skin ulcers caused by pressure, venous stasis, or diabetes mellitus[1]. Diabetes represents a major impact on wound healing outcome. In 2004, according to the World Health Organization (WHO), more than 150 million people worldwide suffered from diabetes. Its incidence is increasing rapidly and it is estimated that by the year 2025, this number will double.

A wound is a disruption of the continuity of a tissue structure. Injury, by surgery or accident, causes destruction of tissue, disruption of blood vessels, and extravasation of blood constituents and hypoxia. Wound healing is a complex, continual process that has three phases: inflammation, a proliferative phase, and tissue remodeling. Basically, wound healing is the result of interactions among cytokines, growth factors, blood and cellular elements, and the extracellular matrix. The cytokines promote healing by various pathways, such as stimulating the production of components of the basement membrane, preventing dehydration, and increasing inflammation and formation of granulation tissue.

At the cellular level, monocytes infiltrate the wound site and become activated macrophages that release growth factors, such as platelet-derived growth factor (PDGF) and vascular endothelial growth factor (VEGF), which initiate the formation of granulation tissue. Macrophages have a key role in inflammation and repair[2]. It has been found that macrophage-depleted animals have defective wound repair[3]. We have found that macrophage transfusion accelerates wound healing in patients with nonhealing wounds[4]. Platelets facilitate the formation of a hemostatic plug and secrete PDGF, which attracts and activates macrophages and fibroblasts[2]. Re-epithelialization of wounds begins shortly after injury. Epidermal cells at the wound margin begin to proliferate within 1 to 2 days after injury. On day 4 after injury, new granulation tissue begins to invade the wound gap and numerous new capillaries grow through the new stroma with its granular appearance. After migrating into wounds, fibroblasts begin the synthesis of the extracellular matrix[2,5].

The induction of angiogenesis was initially attributed to acidic or basic fibroblast growth factors, which are released from macrophages after cell disruption. Angiogenesis is the process of new vessel formation from an existing vasculature network. Once the wound is filled with new granulation tissue, angiogenesis ceases and many of the new blood vessels disintegrate as a result of apoptosis[6]. Wound contraction involves a complex interaction of cells, extracellular matrix, and cytokines.

Vitamins C, E, and A, glucose, amino acids, antioxidants, fatty acids, proteins, water, and zinc are important for wound healing[7,8,9,10,11,12]. Administration of ascorbic acid protected mice against radiation-induced sickness and mortality, and improved healing of wounds after exposure to whole-body gamma radiation[13]. Low levels of antioxidants accompanied by raised levels of markers of free radical damage play a significant role in the delay of wound healing. In diabetic rats, reduced glutathione levels had a role in delaying the healing process[14]. Hydrogen peroxide is one of the mediators of healing responses[15]. Electrolyzed, strong acid aqueous solution irrigation may promote tissue growth in burn wounds[16]. Acidic media enhances wound contraction[7].

Nitric oxide (NO) has a wide range of physiological and pathophysiological activities, including the regulation of vessel tone and angiogenesis in wound healing, inflammation, ischemic cardiovascular diseases, and malignant diseases[17]. NO has been shown to increase microcirculatory blood flow, to kill infective organisms, and to have a significant effect in promoting wound healing[18,19,20,21,22,23]. Prostaglandins are mediators of inflammation and smooth-muscle stimulants, but inhibition of the prostaglandins and their precursors failed to alter the course of wound contraction[24].

Acute or chronic wounds can usually be covered by synthetic or natural dressings. Conservative methods of wound care include the use of standard wound dressings, management of underlying problems (such as hyperglycemia), debridement of dead tissue, restoration of adequate tissue perfusion, limitation of pressure at the wound site, and control of infection. These methods are successful in the majority of patients with acute or chronic skin wounds. However, large and life-threatening skin wounds may require the use of cultured, autologous, epidermal-cell grafts or biologic skin substitutes.

Recombinant PDGF has been approved by the U.S. Food and Drug Administration (FDA) for the treatment of wounds. Furthermore, topical treatment of wounds is an important aspect of wound care. Proper selection of antiseptic or antimicrobial treatment for contaminated wounds is a cornerstone of wound care. Five commonly used topical agents are 5% mafenide acetate, 10% povidone with 1% free iodine, 0.25% sodium hypochlorite, 3% hydrogen peroxide, and 0.25% acetic acid[25]. In addition, hyaluronic acid was used for wound dressing[26]. Basically, modulation of nutrition, underlining medical problems (such as vascular and neural diseases, diabetes, and infection), acidity of wounds, host immunity, cytokines, NO, or prostaglandins have a great impact on wound healing process.

### HEALING PROPERTIES OF HONEY

Honey has long been documented as having healing properties[27,28,29]. Honey and sugar paste were associated with scarless healing in cavity wounds[30]. It has been reported that rabbit wounds treated with a topical application of honey showed less edema, fewer polymorphonuclear and mononuclear cell infiltrations, less necrosis, better wound contraction, improved epithelialization, and lower glycosaminoglycan and proteoglycan concentrations[31]. Furthermore, honey causes significantly greater wound contraction than controls, and it promotes the formation of granulation tissue and epithelialization of wounds[32,33,34,35,36,37]. Honey stimulates tissue growth, synthesis of collagen, and development of new blood vessels in the bed of wounds[38,39,40,41,42,43]. Intraperitoneal honey administration after an adhesion model in the cecum and terminal ileum of rats reduced postoperative adhesion[44].

### HONEY AND WOUNDS

Generally, wound healing can be affected by endogenous (pathophysiology) and exogenous (micro-organisms) factors. The risk of wound infection increases as local conditions favor bacterial invasion and growth. Therefore, microbial colonization of both acute and chronic wounds is inevitable. Many species of bacteria have been recovered from wounds, but *Staphylococcus aureus* is the most frequently isolated from wound pathogens[45]. In addition, *Pseudomonas aeruginosa* is an important pathogen in chronic wounds and burns; its presence has been demonstrated in numerous studies and has been found in one-third of chronic leg ulcers[46,47,48,49]. Infection with *S. aureus* and pseudomonads retards ulcer healing rates and, with pseudomonads and B-hemolytic streptococcus, reduces the success of skin grafts used for leg ulcers[50,51].

The widespread development of antibiotic-resistant bacteria is a challenging problem. Therefore, current interest is focused on an alternative to antibiotics and conventional therapies, such as honey, antimicrobial moisture-retentive dressings, essential oils and cationic peptides, topical enzymes, biosurgical therapies, and vacuum therapies. In addition, unregulated inflammation caused by both micro-organisms and underlying abnormal pathophysiological conditions is a major factor associated with the process of healing in chronic wounds[52].

Many research works reported the use of honey for treatment of both wounds and infections[53,54,55]. Table 1 summarizes many papers that reported successful use of honey in wound healing. Honey with proven antibacterial activity has the potential to be an effective treatment option for wounds infected or at risk of infection with various human pathogens. The medical literature on treating wounds with honey has been reviewed[56,57,58,59,60,61]. As a dressing on wounds, honey provides a moist healing environment, rapidly clears infection, deodorizes, and reduces inflammation, edema, and exudation. It increases the rate of healing by stimulation of angiogenesis, granulation, and epithelialization[62]. Table 2 demonstrates general effects of honey on the healing process.

**TABLE 1**
**Effects of Honey on Wounds Healing**

| Origin of Honey | Type of Lesion | Effects of Honey |
|---|---|---|
| Iran[31] | Surgical incision on rabbits | Less edema, fewer polymorphonuclear and mononuclear cell infiltrations, less necrosis, better wound contraction, improved epithelialization, and lower glycosaminoglycan and proteoglycan concentrations |
| India[39] | Wounds created on buffalo calves | Promotes granulation and scar formation, complete healing of full-thickness wounds occurred faster with honey than with nitrofurazone or sterilized petrolatum |
| India[40] | Wounds created on buffalo calves infected with *S. aureus* | Faster rate of healing compared with ampicillin ointment and saline treatments, the least inflammation, the most rapid fibroblastic and angioblastic activity and epithelialization |
| India[42] | Full-thickness skin wounds made on back of rats | Increases significantly the quantity of collagen synthesized and degree of cross-linking of the collagen in the granulation tissue |
| Egypt[64] | Infected diabetic foot wounds | Fast healing and significant decrease of bacterial load |
| Turkey[65] | Split-thickness skin graft donor site | Wounds show faster epithelialization time and a low sense of pain than paraffin gauzes and saline-soaked gauzes |
| Turkey[44] | An adhesion model constituted in the cecum and terminal ileum of rats | Intraperitoneal honey administration reduces postoperative peritoneal adhesion |
| Turkey[66] | Intraurethral injury in rats | Prevents inflammation, accelerates urethral healing, and provides perfect healing |
| United Arab Emirates[67] | Injured skin or conjunctiva in mice or rat | Accelerates wound healing and eradicates infection |
| Yemen[68] | Postoperative wound | Eradicates bacterial infection, accelerates wound healing, and minimizes scar formation |
| Thailand[69] | Postoperative wound disruption | Complete wound healing within 2 weeks |
| Nepal[71] | Radiation-induced oral mucositis | Strongly protective against the development of mucositis |
| Nigeria[72] | Wounds and ulcers | Debrides wounds rapidly, replacing sloughs with granulation tissue, promotes rapid epithelialization, and absorption of edema |
| Nigeria[73] | Fournier's gangrene | Accelerates wound healing |
| Mexico[33] | Fournier's gangrene | Accelerates wound healing |
| Malawi[76] | Patients with open or infected wounds | More effective than sugar in reducing bacterial contamination and promoting wound healing |
| Norway[80] | Chronic wound infection | Eradicates wound bacterial infections and penetrates biofilm |
| Ireland[81] | Nonhealing ulcers | Manuka honey decreases wound pH and causes a reduction in wound size |
| France[82] | Wounds | Accelerates wound healing |
| Germany[84] | Resistant wound infection in seven patients | Complete wound healing |
| U.K.[85] | Toenail surgery | Partial avulsion wounds healed faster with paraffin tulle gras than with the honey dressing |
| U.K.[88] | Chronic wounds | Clinical benefits from using honey in wound care |
| U.K.[89] | Meningococcal skin lesions | Helps skin healing |
| Netherlands[91] | Sixty patients with chronic wounds | Honey is easy to apply, helpful in cleaning the wounds, and without side effects |

**TABLE 2**
**General Effects of Honey on Wound Healing**

| | |
|---|---|
| 1. | Causes greater wound contraction[31,32,33,34,35,36,37,211]) |
| 2. | Promotes the formation of granulation tissue[32,33,34,35,36,37,39,92] |
| 3. | Promotes epithelialization of wounds[32,33,34,35,36,37,66,73,92] |
| 4. | Stimulates tissue growth, synthesis of collagen[38,39,40,41,42,43,92] |
| 5. | Stimulates development of new blood vessels in the bed of wounds[38,39,40,41,42,43,62,93] |
| 6. | Reduces postoperative adhesion[44] |
| 7. | Reduces edema[62,73,92,93] |
| 8. | Reduces inflammation[61,62,68,92,93,119] |
| 9. | Deodorizes wounds[62,92,93] |
| 10. | Promotes moist wound healing[61,92] |
| 11. | Facilitates debridement[61,72,93] |
| 12. | Reduces pain[66,84,92] |

Molan[63] published a review article that covered the various reports that have been published on the clinical usage of honey. Positive findings on honey in wound care have been reported from 17 randomized controlled trials (1965 patients) and five clinical trials of other forms (97 patients) treated with honey. On experimental animals, the effectiveness of honey in assisting wound healing has also been demonstrated in 16 trials. There is also a large amount of evidence in the form of case studies[63]. In a recent review aimed to determine whether honey increases the rate of healing in acute wounds (burns, lacerations, other traumatic wounds) and chronic wounds (venous ulcers, arterial ulcers, diabetic ulcers, pressure ulcers, infected surgical wounds), 19 trials (n = 2554) were identified. Conclusively, honey may improve healing times in mild to moderate superficial and partial-thickness burns compared with some conventional dressings. However, honey dressings as an adjuvant to compression do not significantly increase leg ulcer healing at 12 weeks[58]. Another article provides an overview of the use of honey in wound management and reviews the evidence to support its effectiveness in the management of wound healing[59]. Honey has anti-inflammatory and antibacterial effects without antibiotic resistance; it promotes moist wound healing and facilitates debridement. A summary of the current evidence base for the use of honey, a review of its therapeutic effects, and a discussion of the implications for WOC nursing practice was published recently[61]. In the U.S., a honey product received FDA approval in 2007.

In Iran, topical application of honey on wounds created on rabbits showed less edema and necrosis, fewer polymorphonuclear and mononuclear cell infiltrations, better wound contraction, improved epithelialization, and lower glycosaminoglycan and proteoglycan concentrations[31].

In India, a study showed that granulation, scar formation, and complete healing of full-thickness wounds created on buffalo calves occurred faster with honey than with nitrofurazone or sterilized petrolatum[39]. In another study, full-thickness skin wounds were made on buffalo calves after infecting the wound by subcutaneous injections of *S. aureus* 2 days prior to wounding. Topical application of honey, ampicillin ointment, and saline were compared. Honey gave a faster rate of healing compared with ampicillin ointment and saline treatments, the least inflammation, the most rapid fibroblastic and angioblastic activity, and epithelialization[40]. Full-thickness skin wounds made on the backs of rats were treated with topical application of honey to the wound, oral administration of honey, or intraperitoneal administration of honey. Honey increased significantly the quantity of collagen synthesized and degree of cross-linking of the collagen in the granulation tissue. Systemic treatment gave greater increase than topical treatment, while the intraperitoneal route produced better results than the oral route[42]. The thickness of granulation tissue and the distance of epithelialization from the edge of the wound were significantly greater and the area of the wound significantly smaller in wounds treated with honey compared with control when honey was applied on excising skin on the mice down to muscles[43].

In Egypt, 30 infected diabetic foot wounds were randomly selected for treatment with clover honey. The honey dressing was applied to wounds for 3 months until healing, grafting, or failure of treatment. Results showed that complete healing was significantly achieved in 43.3% of ulcers, and decrease in size and healthy granulation was significantly observed in another 43.3% of patients. After 1 week, bacterial load of all ulcers was significantly reduced. The authors reported that commercial clover honey is a clinical and cost-effective dressing for diabetic wounds in developing countries[64].

From Turkey, for the treatment of a split-thickness skin graft donor site, honey-impregnated gauze showed faster epithelialization time and a lower sense of pain than paraffin gauzes and saline-soaked gauzes[65]. Another study was performed to evaluate the effect of honey applied intraurethrally after urethral injury on histopathological healing in male rats. Results showed that intraurethral honey, applied after urethral injury, prevents inflammation and accelerates urethral healing[66]. A study has shown that intraperitoneal honey administration reduces postoperative peritoneal adhesion; the wound healing accelerative effect and the mechanical barrier formed by the honey was explained to be the reasons for its inhibitory effect on the adhesion[44].

From the United Arab Emirates, Al-Waili found that topical application of multifloral honey on wounds infected with various human pathogens inoculated onto injured skin or conjuctiva in mice and rats accelerates wound healing and eradicates bacterial infections[67]. Al-Waili and Salom have reported that multifloral honey collected from Yemen applied to postoperative wound infections could eradicate bacterial infections, accelerate wound healing, and minimize scar formation[68].

In Thailand, the usefulness of honey application as an alternative method of managing abdominal wound disruption was assessed. Fifteen patients whose wounds disrupted after caesarean section were treated with honey application and wound approximation by Micropore™ tape instead of the traditional method of wound dressing with subsequent resuturing. Within 2 weeks, an excellent result was achieved in all the cases with complete healing[69].

In Israel, nine infants with large, open, infected wounds that failed to heal with conventional treatment were treated with honey. All infants showed marked clinical improvement after 5 days of treatment with topical application of 5–10 ml of honey twice daily[70].

Another study from Nepal investigated whether honey's anti-inflammatory properties might limit the severity of radiation-induced oral mucositis. A single-blinded, randomized, controlled clinical trial was carried out to compare the mucositis-limiting qualities of honey with lignocaine. It was found that honey is strongly protective against the development of mucositis. The authors concluded that honey applied topically to the oral mucosa of patients undergoing radiation therapy appears to provide a considerable benefit by limiting the severity of mucositis[71].

In Nigeria, 59 patients with wounds and ulcers, most of which had failed to heal with conventional treatment, were treated with unprocessed honey. Fifty-eight cases showed remarkable improvement following topical application of honey. Honey debrided wounds rapidly, replacing sloughs with granulation tissue. It also promoted rapid epithelialization and absorption of edema from around the ulcer margins[72]. In another study from Nigeria, 20 consecutive cases of Fournier's gangrene managed conservatively with systemic antibiotics and topical application of honey were compared retrospectively with 21 similar cases managed by wound debridement and excision[73]. Honey has been compared with phenytoin in a prospective randomized controlled trial on chronic leg ulcers[74]. Two groups of 25 patients with ulcers were involved, with a mean duration of 56.5 months. There was no significant difference found in the rate of healing between the honey and phenytoin treatments.

In Burundi, 40 patients with wounds of various origins were treated with topical honey, which provided healing in 88% of the cases[75]. In Mexico, 38 patients with Fournier's gangrene were treated with antimicrobial therapy, broad debridement, and application of unprocessed honey dressings. Patients then underwent split-thickness skin grafts or delayed closure as needed. It was found that topical application of honey is beneficial to the healing process[33]. Results showed that topical application of honey was better than conventional methods. Three deaths occurred in the orthodox method, whereas no deaths occurred in those treated with honey.

In Malawi, to investigate whether there is a difference between the efficacy of honey and sugar as wound dressings, patients with open or infected wounds were randomized to receive either honey or sugar dressings. Forty patients were enrolled. In the honey group, 55% of patients had positive wound cultures at the start of treatment and 23% at 1 week, compared with 52 and 39%, respectively, in the sugar group. Honey was more effective than sugar in reducing bacterial contamination and promoting wound healing, and was slightly less painful than sugar during dressing changes[76].

From South Africa, the effects of silver- and honey-based dressings on cell viability (keratinocyte cultures) were compared. Results showed that there was no significant difference between the best performing silver- and honey-based wound preparations with regard to cell viability[77]. Another study from the same area was planned to establish whether honey (L-Mesitran®) and silver-impregnated dressings are cytotoxic *in vitro* to human skin keratinocytes and dermal fibroblasts. In cultures with honey-impregnated implants, cell viability remained intact and cell toxicity was not evident at 4 months after continuous tissue culture. In cultures with nanocrystalline silver, marked toxicity was observed with high nonviability staining and cell-scoring counts. The honey-based product showed excellent cytocompatibility with tissue cell cultures compared with the silver dressing[78]. Furthermore, it was found that there was no evidence of a real difference between honey and IntraSite™ Gel as healing agents. It was concluded that honey is a safe, satisfying, and effective healing agent and is cost effective[79].

In Norway, the effects of different concentrations of Medihoney™ therapeutic honey and Norwegian Forest Honey (1) on the real-time growth of typical chronic wound bacteria, (2) on biofilm formation, and (3) on the same bacteria already embedded in biofilm were studied. Reference strains of *MRSE, MRSA, ESBL Klebsiella pneumonia,* and *P. aeruginosa* were incubated with dilution series of the honeys in microtiter plates for 20 h. It was found that both honeys were bactericidal against all the strains of bacteria. Biofilm was penetrated by biocidal substances in the honey[80]. In Ireland, a study was conducted to analyze the changes in surface pH and size of nonhealing ulcers following application of manuka honey dressing. Reduction in wound pH after 2 weeks was statistically significant. Wounds with pH $\geq$8.0 did not decrease in size and wounds with pH $\leq$7.6 had a 30% decrease in size. The use of manuka honey dressings was associated with a statistically significant decrease in wound pH and a reduction in wound size[81].

In France, 40 patients with wounds of various etiology – surgical, accidental, infective, trophic, and burns – were treated with topical application of honey. Of the 33 patients treated only with honey dressing, 29 patients healed successfully. Honey delimited the boundaries of the wounds and cleansed them rapidly[82].

In Sweden, when commercial unboiled honey was applied topically to open wounds in mice, the wounds of the honey-treated animals healed much faster than the wounds of the control animals[43].

In Poland, the case of a 55-year-old woman with extensive phlegmonous and necrotic lesions of the abdominal integuments and the lumbar area following traumatic colonic rupture, treated with manuka honey wound dressings and the GENADYNE A4 negative pressure wound healing system, was discussed. The results showed that this intervention brought good effects, ultimately enabling skin autografting on the wound site and complete wound healing[83].

In Germany, full healing was achieved in seven patients whose wounds were either infected or colonized with MRSA. Antiseptics and antibiotics had previously failed to eradicate the clinical signs of infection[84].

In the U.K., honey-impregnated dressings successfully were used in the wound care clinic and on the maxillofacial ward[85]. A double-blind, randomized controlled trial investigated the effect of a honey dressing on wound healing following toenail surgery with matrix phenolization. One hundred patients were randomly assigned to receive either an active manuka honey dressing or paraffin-impregnated tulle gras. Mean healing times were 40.30 days for the honey group and 39.98 days for the paraffin tulle gras group. Partial avulsion wounds healed statistically significantly faster with paraffin tulle gras than with the honey dressing[86]. In another report, a case study explored the healing of a chronic wound (20 years' duration) in a patient with dystrophic epidermolysis bullosa with use of a honey-impregnated dressing; the wounds were not responding to different dressings and creams[87]. Another study from the U.K. that was conducted to compare a medical-grade honey with conventional treatments on the healing rates of wounds healing by secondary intention included 105 patients who were involved in a single-center, open-label, randomized controlled trial in which patients received either a conventional wound dressing or honey. The median time to healing in the honey group was 100 days compared with 140 days in the control group. The authors concluded that there are clinical benefits from using honey in wound care[88]. Chronic infected meningogococcal skin lesions were successfully treated with honey[89]. In another study from the same country, manuka honey was found to eradicate MRSA in a hydroxyurea-induced leg ulcer developed in an immunosuppressed patient; rapid healing was successfully achieved[90].

In the Netherlands, authors evaluated the use and safety of a honey-medicated dressing in a feasibility (phase II) study featuring 60 patients with chronic, complicated, surgical, or acute traumatic wounds. In all but one patient, honey was found easy to apply, helpful in cleaning the wounds, and without side effects[91].

Generally, it has been shown that topical honey possesses antimicrobial properties, promotes autolytic debridement, stimulates growth of wound tissues to hasten healing and to start the healing process in dormant wounds, stimulating anti-inflammatory activity that rapidly reduces pain, edema, and exudate production[92]. Patients with recalcitrant wounds and ulcers, including Fournier’s gangrene, burns, cancrum oris and diabetic ulcers, decubitus, sickle-cell and tropical ulcers, were successfully treated with topical honey application; all had been treated for a long time with conventional treatment with no signs of healing or with the wounds increasing in size[93]. Basically, there have been many reports of case studies, animal experiments, and randomized controlled trials that provide considerable evidence for the effectiveness of honey in wound healing. As a dressing on wounds, honey provides a moist healing environment, rapidly clears infection, deodorizes, and reduces inflammation, edema, and exudation.

### HONEY AND SKIN ULCERS

Honey has been used in the treatment of ulcers due to various etiologies[42,94,95,96,97,98,99,100,101]. In a review of the literature, more than 470 cases were treated with honey; there were only five cases where successful healing was not achieved[102]. In another review study, the authors summarized evidence of honey’s effectiveness, its hypothesized mechanism of action, potential risks and benefits, the types of honey available, and the nature of its application. Critical aspects of ulcer care are also reviewed. It was concluded that honey is a low-cost topical therapy with important potential for healing[103]. The use of honey in two patients with pressure ulcers resulted in a rapid and complete healing of both wounds. The antibacterial activity of honey had a deodorizing effect on the wounds and its anti-inflammatory actions reduced the level of pain[95]. Further, honey was used successfully for necrotizing breast ulcer management[101]. Out of 59 treated patients with wounds and ulcers, 58 patients showed remarkable improvement following topical application of honey; sloughs, necrotic, and gangrenous tissue separated and could thus be lifted off painlessly[73].

In the U.K., three patients with chronic leg ulceration were assessed as potentially benefiting from the action of medical honey to achieve wound healing. The etiology of ulceration in patient 1 was mixed arterial and venous, and in patients 2 and 3, venous. All had several years’ history of recurrence. Promotion of healing occurred in all the cases, with a reduction in the incidence of infection, reduction in pain, and the provision of comfort[104]. In Malaysia, authors carried out a prospective study to compare the effect of honey dressing for 30 Wagner’s grade-II diabetic foot ulcers with a controlled dressing group (povidone iodine followed by normal saline). Surgical debridement and appropriate antibiotics were prescribed for all patients. The mean healing time in the standard dressing group was 15.4 days compared to 14.4 days in the honey group; ulcer healing was not significantly different in both study groups. It was concluded that honey dressing is a safe, alternative dressing for Wagner’s grade-II diabetic foot ulcers[105]. In another study, 40 patients whose leg ulcers had not responded to 12 weeks of compression therapy were recruited. Medihoney dressings were applied on their ulcers for the 12-week study period; ulcer pain and size decreased significantly and odorous wounds were deodorized promptly[106].

In Ireland, a study was done to determine the qualitative bacteriological changes that occurred during a 4-week treatment period with either manuka honey or a hydrogel dressing; 108 patients enrolled in the study. At baseline, MRSA was identified in 16 wounds (10 in the honey group vs. six in the hydrogel group). Results showed that manuka honey was effective in eradicating MRSA from 70% of chronic venous ulcers. The potential to prevent infection is increased when wounds are desloughed and MRSA is eliminated[107]. In another report, three males and five females with leg ulceration of different etiologies were treated with honey. The wounds were dressed weekly with manuka honey. The size of the ulcers was significantly reduced, the odor was eliminated, and the pain was relieved. The authors concluded that the use of manuka honey was associated with a positive wound healing outcome[108].

In Turkey, a 5-week, randomized clinical trial evaluated the effect of a honey dressing on pressure ulcer healing, and comparison with an ethoxy-diaminoacridine plus nitrofurazone dressing was conducted. Thirty-six patients with a total of 68 stage II or III pressure were enrolled in the study. After 5 weeks of treatment, patients who were treated by honey dressing had significantly better PUSH tool scores than subjects treated with the ethoxy-diaminoacridine plus nitrofurazone dressing[109].

In Pakistan, a study was carried out to export the results of topical wound dressings in diabetic wounds with natural honey. Initially, all wounds were washed thoroughly, necrotic tissues were removed, and dressings with honey were applied. The study demonstrated excellent results in treating diabetic wounds with dressings soaked with natural honey. The disability of diabetic foot patients was minimized by decreasing the rate of leg or foot amputations and, thus, enhancing the quality and productivity of individual life[110].

Contrary to previous works, a community-based, open-label, randomized trial allocated people with venous ulcers to calcium alginate dressings impregnated with manuka honey or usual care. Of 368 participants, 187 were randomized to honey and 181 to usual care. At 12 weeks, 104 ulcers in the honey-treated group and 90 in the usual care group had healed. The authors stated that the treatment with honey was probably more expensive and associated with more adverse events. It was concluded that honey-impregnated dressings did not significantly improve venous ulcer healing at 12 weeks compared with usual care[111]. In Nigeria, the efficacy of locally applied natural honey was compared to that of Eusol solution in an open trial involving 20 patients with sickle cell anemia who were in steady clinical states and had chronic leg ulceration. Eleven ulcers were dressed with honey, while eight were dressed with Eusol. No significant differences were found in the rates of healing of the ulcers in either treatment groups[112].

### HONEY AND BURNS

Burn injuries are associated with a high incidence of death and disability. Advances in cellular biology, and knowledge in wound healing and growth factors, have helped to move steps toward burn management. Split-thickness skin grafting with autografts is the standard of care. Honey was used for management of burns and the use of honey for burns has been reviewed[113,114,115,116,117,118]. Various modalities have been reviewed, including honey, human amnion, xenograft, allograft, cultured epithelial autograft, and various engineered commercial products, for use in the biologic treatment of burn wounds[116]. A systematic review and meta-analysis of randomized controlled trials that compared the efficacy of honey with a comparator dressing treatment in the management of burns was conducted. Eight studies with 624 subjects were included. It was found that in most studies, honey covered by sterile gauze was compared with silver sulfadiazine–impregnated gauze. The fixed effects odds ratio for healing at 15 days was 6.1 (95% CI 3.7–9.9) in favor of honey having a superior effect. The secondary outcome variables all showed significantly greater efficacy for honey treatment. It was concluded that available evidence indicates markedly greater efficacy of honey compared with alternative dressing treatments for superficial or partial-thickness burns[118].

In the U.K., a review study aimed to investigate topical honey for superficial burns through a systematic review of randomized controlled trials. Five studies in patients with partial-thickness or superficial burns involving <40% of the body surface were reviewed. Comparators were polyurethane film, amniotic membrane, potato peel, and silver sulfadiazine. The number needed to treat for 7 days with honey to produce one patient with a healed burn was 2.6 (2.1–3.4) compared with any other treatment, and 2.7 (2.0–4.1) compared with potato peel and amniotic membrane. For most of the outcomes, honey was superior to all other treatments. In addition, time for healing was significantly shorter for honey than other treatments[117]. In France, it was found that healing was rapid for first- and second-degree burns[119]. Honey produced attenuation of inflammation and exudation, plus rapid regeneration of outer epithelial tissue and cicatrization. In the Netherlands, honey-treated burns showed less inflammation than those treated with sugar and silver sulfadiazine[120].

In Malaysia, full-thickness burn wounds created on the dorsum of 36 rats were inoculated with *P. aeruginosa, K. pneumoniae,* or *Acinetobacter baumannii*. The wounds were dressed with tualang honey, hydrofiber, and hydrofiber silver. Results showed that there was a rapid reduction in wound size by day 6 in the tualang honey–treated wounds. Tualang honey–treated rats demonstrated a reduction in bacterial growth in *P. aeruginosa*–inoculated wounds. However, hydrofiber silver– and hydrofiber-treated wounds are superior to honey-treated wounds with *A. baumannii*[121]. Antibacterial activity of Aquacel-tualang honey, Aquacel-manuka honey, Aquacel-Ag, and Aquacel-plain dressings against bacteria isolated from burn patients were tested *in vitr*o. Seven organisms were isolated: *Enterobacter cloacae, K. pneumoniae, Pseudomonas* spp., *Acinetobacter* spp., *S. aureus,* coagulase-negative *S. aureus,* and *Streptococcus* spp. Aquacel-Ag and Aquacel-manuka honey dressings gave a better zone of inhibition for Gram-positive bacteria compared to the Aquacel-tualang honey dressing. However, comparable results were obtained against Gram-negative bacteria tested with Aquacel-manuka honey and Aquacel-tualang honey dressings. Tualang honey has a bactericidal as well as bacteriostatic effect[122].

In Pakistan, in a randomized, comparative clinical trial, the efficacy of honey for the treatment of superficial and partial-thickness burns covering <40% of body surface area was evaluated in 150 patients and compared its results with those of silver sulfadiazine. The rate of re-epithelialization and healing of superficial and partial-thickness burns was significantly faster in the sites treated with honey than in the sites treated with silver sulfadiazine. Six patients had positive culture for *P. aeruginosa* in the honey-treated site, whereas 27 patients had positive culture in the silver sulfadiazine–treated site[123].

Another study was carried out in Iran to compare the effect of honey and mafenide acetate on auricular burn in rabbits. Results showed that although the pathologic score of the honey group was better than that of the mafenide group, both on 14 and 21 days after burning, it was not statistically significant. In the mafenide acetate group, deep complication of burn (chondritis) was significantly lower than that of the honey group[124].

In Turkey, a study evaluated honey use for the split-thickness skin graft fixation because of its adhesive and other beneficial effects in 11 patients. No complications, such as graft loss, infection, or graft rejection, were reported[125].

In India, a prospective, randomized clinical study to compare honey-impregnated gauze with amniotic membrane dressing in partial-thickness burns was performed. Sixty-four patients were studied; 40 were treated with honey-impregnated gauze and 24 were treated with amniotic membrane. The burns treated with honey healed earlier as compared to those treated with the amniotic membrane (mean 9.4 vs. 17.5 days). Residual scars were noted in 8% of patients treated with honey-impregnated gauze and in 16.6% of cases treated with amniotic membrane[126]. In another study, 104 cases of superficial burn injury were treated with honey as a dressing or with silver sulfadiazine gauze dressing. In the 52 patients treated with honey, 91% of wounds were rendered sterile within 7 days. In the 52 patients treated with silver sulfadiazine, 7% showed control of infection within 7 days. In addition, healthy granulation tissue was observed earlier in patients treated with honey, and of the wounds treated with honey, 87% healed within 15 days as compared to 10% in the control group[113]. In India, the effect of honey dressing and silver sulfadiazine dressing on wound healing in burn patients was studied in 78 patients with first- and second-degree burns of <50% of total body surface area. Honey dressings improved wound healing, made the wound sterile in less time, had a better outcome in terms of prevention of hypertrophic scarring and postburn contractures, and decreased the need of debridement irrespective of time of admission, when compared to silver sulfadiazine dressings[127].

Twenty-five patients with burns were randomly allocated for treatment with honey or silver sulfadiazine. Of the wounds treated with honey, 84% showed satisfactory epithelialization by the 7th day and 100% of the patients by the 21st day. In addition, in honey-dressed wounds, early subsidence of acute inflammatory changes, better control of infection, and quicker wound healing was observed[38]. Honey-impregnated gauze was compared with OpSite™ as a cover for fresh, partial-thickness burns in two groups of 46 randomly allocated patients. Results showed that wounds dressed with honey-impregnated gauze showed earlier healing as compared those dressed with OpSite[127].

### HONEY AND WOUND DRESSING

Honey has been used for its healing properties for centuries and has been used to dress wounds with promising results. Honey dressings increase healing, minimize debridement, prompt successful graft, remove dry crust, prevent dry scab formation on burns, cleanse wounds, ease separation of sloughs, deodorize wounds, cause soothing of wounds, and minimize scar formation[33,38,41,73,75, 126,127,128,129,130,131]. Honey dressings are easy to apply and remove[73,128,129,130,131,132]. The use of honey as a wound dressing was reviewed recently[133].

Honey has antibacterial activity and, consequently, it is effective in treating infected wounds[43,44,45,46,47,48,49,50,51,52,53,54,55,56,57,58,59,60,61,62,63,64,65,66,67,68,69,70,71,72,76, 77,132,134,135,136,137,138,139,140,141,142,143,144,145,146,147,148,149,150]. Clinical studies on the use of honey as a dressing for infected wounds report that the wounds become sterilized in 3–10 days[100,101,102]. Al-Waili and Salom have used honey dressings for treatment of postoperative caesarean section wound infections[68]. Honey applied to the wounds after washing with normal saline and covered with dry gauze reduced healing time and hospital stay, promoted fast eradication of bacterial infection, prevented wound dehiscence, and caused minimal scar formation. Honey could act as a barrier, which would prevent wound infection[38,72,132]. In clinical practice, honey is spread on the wound, then covered with dry gauze[38,69,75]. In case of abscess or necrotic tissue, honey is applied after drainage of abscess or removal of necrotic tissues[33,151].

### MECHANISMS OF ACTION

Wound infection is an important factor that delays or inhibits wound healing. Wound healing needs a good healthy environment so that the normal physiological process will result in a normal healing process with minimal scar formation. One of the most important strategies to keep the process of healing ongoing is to sterilize damaged tissue from any microbial infection. Honey has many effects, such as antibacterial, antioxidant, antitumor, anti-inflammatory, and various metabolic effects. Regarding antibacterial activity, inhibition of bacterial growth has been shown using impregnated honey disks or incorporating honey into agar plates[152]. How much of this inhibition is due to honey’s antimicrobial properties or to its acidity and hyperosmolar nature is not well established[152]. In this regard, the hyperosmolar sugar paste also has antibacterial activity and is superior to antiseptics[153]. Various studies explore that honey has beneficial effects on wound healing apart from its antibacterial properties. The accelerative effect of honey in the wound healing process is related to its physical properties of hygroscopicity, hypertonicity, lower pH, and complex chemical composition. However, the stimulatory effects obtained when honey was administered orally or parenterally suggest that a tissue growth factor may be involved, rather than stimulation of growth being a consequence of wound acidification or improved nutrition of the tissue.

### Hydrogen Peroxide

Hydrogen peroxide ($H_2O_2$) is important as an antiseptic and stimulates the wound healing process. Recent work using zebrafish reveals a novel mechanism of early leukocyte recruitment to wounds through a concentration gradient of $H_2O_2$[154]. Neutrophils release bactericidal reactive oxygen species and $H_2O_2$ to kill bacteria and prevent infection. Macrophages arrive at the wound in response to environmental stimuli and release VEGF, an angiogenic factor crucial for wound healing. $H_2O_2$ increases macrophage VEGF through an oxidant induction of the VEGF promoter. This oxidant stimulation can be mediated by activated neutrophils[155].

It has been assumed that the antibacterial activity of honey is due to $H_2O_2$[156]. $H_2O_2$ is an oxidizing agent released by the action of the enzyme oxidase that is added by bees to nectar[156]. Much of the literature indicates that rates of $H_2O_2$ production by glucose oxidase in honey vary greatly and increase disproportionately with different degrees of honey dilution[157,158,159,160]. $H_2O_2$ is generated on dilution of honey[161]. The concentration of $H_2O_2$ produced in honey is about 1000 times less than in the 3% solution commonly used as an antiseptic[56]. The rate of $H_2O_2$ production per milliliter of honey solution decreased at higher honey concentrations[162]. When honey and sugar are used as dressings, they become diluted[163]. $H_2O_2$ has been reported to stimulate fibroblast proliferation *in vitro* and angiogenesis *in vivo*[164]. Interestingly, honey has high levels of antioxidants that would protect wound tissues from oxygen radicals that may be produced by $H_2O_2$[165].

In Canada, it was suggested that the antimicrobial activity in some honeys depends on the endogenous $H_2O_2$ content. The study was aimed to determine whether honey's $H_2O_2$ level could serve as a honey-specific, activity-associated biomarker that would allow the prediction and assessment of the therapeutic effects of honey. Using a broth microdilution assay, antibacterial activities of 42 Canadian honeys against two bacterial strains, *Escherichia coli* (ATCC 14948) and *Bacillus subtilis* (ATCC 6633), were analyzed. The results show that all Canadian honeys exhibited antibacterial activity, with higher selectivity against *E. coli* than *B. subtilis*, and that these antibacterial activities were correlated with $H_2O_2$ production in honey[166]. Obviously, $H_2O_2$ generated from honey application could help to control wound infection as well as to help wound healing.

### Osmolarity

It was found that solutions of high osmolarity, such as honey, glucose, and sugar pastes, inhibit microbial growth because the sugar molecules tie up water molecules so that bacteria have insufficient water to grow[163]. Therefore, high osmolarity is valuable in the treatment of infections because it prevents the growth of bacteria and encourages healing[153]. Sugar was used to enhance wound healing for several hundred patients[167]. It has been claimed that the sugar content of honey is responsible for its antibacterial activity, which is due entirely to the osmotic effect of its high sugar content[98,132,168,169]. High osmolarity can safely be achieved topically by the use of sugar paste or honey.

### Nonperoxide Activity and Antioxidants

It was found that antibacterial activity persist in honeys treated with catalase to remove the $H_2O_2$ activity[170,171,172,173]. Manuka honey was found to have an exceptionally high level of nonperoxide antibacterial activity[173]. Some floral sources provide additional antibacterial components by way of plant-derived chemicals in the nectar, such as flavonoids and aromatic acids[150]. Oxygen radicals are involved in various aspects of inflammation. Free radicals cause inflammation and damage to tissue[174,175,176,177,178]. The application of antioxidants to burns has been shown to reduce inflammation[1]. Honey inactivates the free iron, which catalyzes the formation of oxygen free radicals produced by $H_2O_2$, and its antioxidant components help to mop up oxygen free radicals[165,179]. Darker honeys were more inhibitory than light-colored honeys. Since antimicrobial activity of the darker honeys was not eliminated by catalase, nonperoxide components, such as antioxidants, may contribute to controlling the growth of some food-borne pathogens[150].

### Physical Properties

The physical properties of honey provide a protective barrier and, by osmosis, create a moist wound healing environment that does not stick to the underlying wound tissues.

### Increased Lymphocytic and Phagocytic Activity

In the U.K., a study was carried out to investigate the effect of each of three honeys (manuka, pasture, and jelly bush) on the release of inflammatory cytokines from MM6 cells. These honeys, together with a sugar syrup control (artificial honey), were incubated with MM6 cells. All honeys significantly increased the TNF-α, IL-1β, and IL-6 release from MM6 cells (and human monocytes) when compared with untreated and artificial honey–treated cells. Jelly bush honey significantly induced the maximal release of each cytokine compared with manuka, pasture, or artificial honeys. These results suggest that the effect of honey on wound healing may in part be related to the stimulation of inflammatory cytokines from monocytic cells. Such cell types are known to play an important role in healing and tissue repair[180]. Honey stimulates proliferation and activation of peripheral blood B and T lymphocytes in cell culture. Jungle honey was found to enhance the immune system and possess chemotactic activity for neutrophils having antitumor activity; the effective immune component of jungle honey is substrate of molecular weight 261[181].

### Acidity

Chronic nonhealing wounds have an elevated alkaline environment. Lowering the wound pH can potentially reduce protease activity, increase fibroblast activity, and increase oxygen release, consequently aiding wound healing. The glucose content of honey and the acid pH may assist in the bacterial-killing action of macrophages[182]. We have found that honey activity increased in acidic media[68]. However, the antibacterial activity was not significantly suppressed upon neutralization of its acidity[68]. In addition, many bacteria and fungi can survive or resist acidic media and can tolerate extremely acidic conditions[183,184,185,186,187]. Therefore, as we previously demonstrated, the acidity of honey could not be a sole factor for its antibacterial activity[68].

In Ireland, a study was conducted to analyze the changes in surface pH and size of nonhealing ulcers following application of a manuka honey dressing after 2 weeks. Wounds with pH $\geq 8.0$ did not decrease in size and wounds with pH $\leq 7.6$ had a 30% decrease in size. The use of manuka honey dressings was associated with a statistically significant decrease in wound pH and a reduction in wound size[82]. It was found that topical acidification of experimental deep partial-thickness burns promoted healing[188].Therefore, honey acidity might help wound healing.

### Nitric Oxide

NO is important for healing, bacterial killing, viral inhibition, immunological response, and respiratory, renal, cardiovascular, and nervous systems functions. Investigators have implicated NO in the inflammatory and proliferative phases of wound healing[189]. Wound healing involves platelet, inflammatory cell, fibroblast, and epithelial cells; all of them are capable of producing NO[190]. NO can reverse impaired healing in diabetic patients[191]. Moreover, NO can enhance bone healing, and it inhibits tumor growth and metastasis[192,193].

It has been shown that NO plays a role in the host defense against various infections[191]. Killing of intracellular pathogens is mediated by NO[192]. Replication of many viruses can be inhibited by NO[194]. It was found that NO is a very important mediator of immune responses and could prevent skin damage induced by UVB[194,195]. We have found that honey contains NO end products[196]. In addition, honey increases NO end products in various biological fluids, such as urine, saliva, and plasma[197,198]. Intravenous honey increased NO end products in plasma and urine[196]. We have found that antimicrobial activity of honey was decreased by exposure to heating and prolong storage[68]. Heating and prolonged storage decreased NO metabolites identified in various kinds of honey. In addition, the concentration of NO metabolites varies in various types of honey. This might help to explain the fact that the antimicrobial activity of honey varies markedly with its origin[199]. Furthermore, it was suggested that many effects of honey on immunity, bacterial infections, and wound healing could be explained by the ability of honey to increase NO production[196,197,198].

### Prostaglandins

Prostaglandins are mediators of inflammation and pain. They are widely regarded as immunosuppressive, which can decrease many aspects of B- and T-lymphocyte functions[200]. They are effective in inhibiting antibody production by B lymphocytes and they increased the induction of a specific T suppressor[201,202]. Prostaglandins reduce immunity and play a critical role in cancer development[203].

Dr. Al-Waili was the first who reported the immunosuppressive effects of prostaglandins on antibody production and, consequently, prostaglandin synthesis inhibitors used to combat tumors and increase immunity[204,205,206,207]. In addition, prostaglandins change serum protein components during antigen stimulation[208]. We have found that honey can lower plasma prostaglandin concentrations in normal individuals[209]. Its inhibitory effect was increased with time. The site of actions could be either at COX-1 or COX-2, or both. Recently, it was found that artificial honey made of glucose and fructose increased prostaglandin concentrations[209]. Therefore, natural honey might contain raw materials that are capable of inhibiting prostaglandin synthesis[209]. The ability of honey to lower prostaglandin concentrations could explain many of its biological and therapeutic effects, particularly those related to inflammation, pain, immunity, and wound healing.

### Antibody Production

It has been demonstrated that honey increased antibody production during primary and secondary immune responses against thymus-dependent and thymus-independent antigens. The actual mechanism to stimulate antibody production was not identified. NO is an important mediator of immune responses[193]. A single dose of L-arginine, a known precursor of NO, caused a significant increase in humoral response[210]. Therefore, honey might increase humoral immunity by means of its ability to enhance NO production. Prostaglandins are immunosuppressive; they are found to inhibit antibody production by B lymphocytes and to increase the induction of T suppressor cells[201,202]. As honey lowers prostaglandin concentrations, the enhancing effect of honey on antibody production was suggested to be attributed to its ability to inhibit prostaglandins[208].

### Nutritional Composition

Honey contains defined substances, such as glucose, fructose, sucrose, minerals, vitamins, antioxidants, amino acids, and other products. Its wide range of activity raised the possibility of the presence of other unidentified substances. The composition with the natural proportion of each substance in the honey might play a significant role in the mechanism of its action and potency. More investigations are needed in order to disclose the possible presence of other natural materials in honey that might play a role in exhibition of its wide biological and therapeutic effects.

### Wound Contracture

In Nigeria, dressing wounds of rats with honey significantly enhanced percentage wound contraction on day 10 with a value of 79.20 ± 2.94 compared to the control value of 53.50 ± 4.32. There was no significant difference in fibroblast count per high power field in the honey group compared to the control group. In addition, honey dressing increased mean blood vessel count per high power field. Also, honey dressing caused increased granulation tissue formation in wounds compared to those of the control group[211].

## CONCLUSION

There are tremendous data supporting the effectiveness of honey in the management of wounds. The data clearly demonstrate that with the use of honey, no allergic reaction is elicited and no significant side effects were reported, and there is rapid elimination of wound odor, improvement of granulation and epithelialization, reduction of amount of exudates, and sterilization of wounds from microbes. In addition to its valuable nutritional constituents, honey has anti-inflammatory and antioxidant activities that make it a suitable natural subject for wound healing. Honey increases NO, which is important for wound healing, and decreases prostaglandins, which are mediators of inflammation. Honey's acidity and osmolarity play an important role in the healing process. Its antioxidant contents are important as wound healers and help in the eradication of microbial infections. In addition, honey has considerable effects on the cellular elements of immunity and antibody production. Although most studies on honey did not mention its plant source, honeys collected from various geographical areas share a similar ability to help both wound healing and microbial control.

The antibacterial, anti-inflammatory, antioxidant, as well as nutritional and physical properties of honey, make it a logical and accepted natural agent for wound dressing.
